# From UK-2A to florylpicoxamid: Active learning to identify a mimic of a macrocyclic natural product

**DOI:** 10.1007/s10822-024-00555-3

**Published:** 2024-04-17

**Authors:** Ann E. Cleves, Ajay N. Jain, David A. Demeter, Zachary A. Buchan, Jeremy Wilmot, Erin N. Hancock

**Affiliations:** 1 BioPharmics Division, Optibrium Limited, Cambridge, CB25 9GL UK; 2 Corteva Agriscience, Indianapolis, IN 46268 USA

**Keywords:** Active-learning, QuanSA, Affinity prediction, Macrocycles

## Abstract

Scaffold replacement as part of an optimization process that requires maintenance of potency, desirable biodistribution, metabolic stability, and considerations of synthesis at very large scale is a complex challenge. Here, we consider a set of over 1000 time-stamped compounds, beginning with a macrocyclic natural-product lead and ending with a broad-spectrum crop anti-fungal. We demonstrate the application of the QuanSA 3D-QSAR method employing an active learning procedure that combines two types of molecular selection. The first identifies compounds predicted to be most active of those most likely to be well-covered by the model. The second identifies compounds predicted to be most *informative* based on exhibiting low predicted activity but showing high 3D similarity to a highly active nearest-neighbor training molecule. Beginning with just 100 compounds, using a deterministic and automatic procedure, five rounds of 20-compound selection and model refinement identifies the binding metabolic form of florylpicoxamid. We show how iterative refinement broadens the domain of applicability of the successive models while also enhancing predictive accuracy. We also demonstrate how a simple method requiring very sparse data can be used to generate relevant ideas for synthetic candidates.

## Introduction

**Fig. 1:**
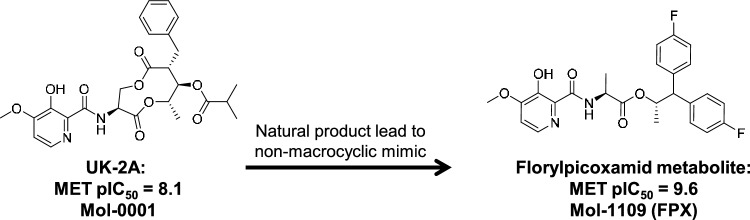
The starting natural product UK-2A is shown (left) along with the binding metabolic form of florylpicoxamid, the final crop protection fungicide

Natural products (NPs) have been used as inspiration for crop protection active ingredients. However, it is often the case that structural features of NPs, such as macrocycles and multiple chiral centers, limit their use due to the expense of industrial-scale synthesis. Figure 1 shows the structure of UK-2A (left side), a natural product with excellent in vitro inhibition of mitochondrial electron transport (MET) complex III via binding to the Q_i_ site of cytochrome *b* [1]. Activity values were determined by an in vitro MET binding assay and expressed here as pIC_50_. Protection of the 3-pyridinol with an isobuytryloxymethyl group improved *in planta* antifungal performance, with the unprotected binding metabolite being readily produced. Figure 1 shows the unprotected form of florylpicoxamid (right side, “FPX”), whose 3-pyridinol protected precursor has been shown to be a highly effective crop protection fungicide [1]. FPX has two fewer chiral centers, no macrocycle, and is fully synthetic, not requiring starting materials from fermentation processes. The development of FPX followed a design strategy of stepwise deconstruction of a macrocyclic natural product, requiring many hundreds of synthetic analogs along with in vitro and *in planta* assays.

Here, we investigate the degree to which an active-learning approach for activity prediction could be used to vastly reduce the number of synthetic analogs required in such an effort. Ligand activity prediction continues to be a challenge for computer-aided drug design, especially in the case where there is no suitable high-resolution experimental structure of the target of interest, as is the case here. An additional challenge here is the presence of flexible macrocyclic ligands. Over the past several years, methods for computational modeling of macrocyclic ligands have made significant progress [2–7]. In particular, natural-product based and semi-synthetic macrocycles of up to roughly 21–23 total rotatable bonds (including both macrocyclic bonds and exocyclic bonds) have been shown to be tractable, in terms of accuracy and speed of conformational search when utilizing multiple computing-cores [7]. However, larger peptidic macrocycles remain challenging, often requiring biophysical data (e.g. from NMR) to help restrain the conformational space to be explored [8]. Generally, the macrocycles studied here fell well within the tractable range of the ForceGen methodology [7].

Machine learning approaches have seen a recent resurgence in their applications within the CADD field, in part driven by advances in deep-learning methodologies. A recent review highlights a number of successful applications as well as limitations [9], with further context provided by a full book treatment [10]. With respect to binding affinity prediction in the context of lead optimization, a critical factor is that such methods typically require thousands of data points in order to learn effectively, because of the need to develop encoded internal representations that meaningfully capture the important aspects required for prediction. Early-stage lead optimization may involve just dozens of assayed molecules within a newly discovered chemical series, and even mid- to late-stage projects may be limited to hundreds or up to a few thousand data points. The recently introduced QuanSA machine-learning method (*Quan*titative *S*urface-field *A*nalysis) differs from the deep-learning paradigm and from historically widely used methods [11, 12] in ways that make it applicable even in early-stage lead optimization.

**Fig. 2:**
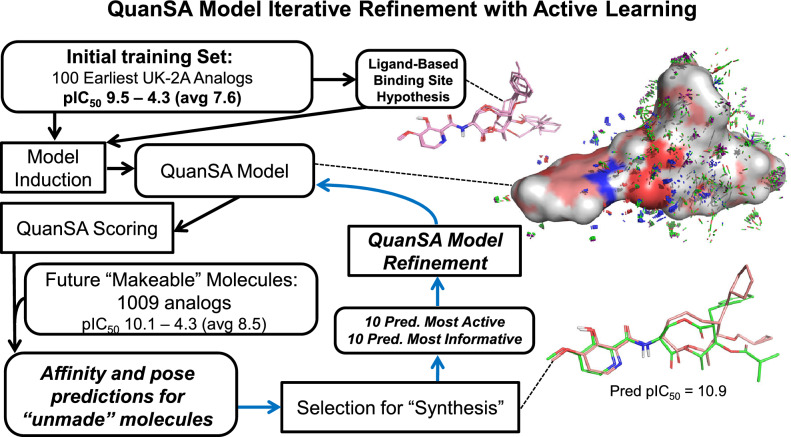
Scheme for iterative model refinement using temporally sorted structure-activity data from lead optimization

The central difference is that rather than applying a generic machine-learning approach to an input molecular representation divorced from a binding event, QuanSA builds a physically interpretable model that is analogous to a protein binding site. By doing so, it addresses the problem of ligand conformation and alignment fully automatically, and it moves in the direction of causal modeling, where the requirement for training data can be reduced. The method constructs a non-linear “pocket-field” that is still physical in nature, and which is directly related to the functional form of scoring functions for docking [13, 14]. QuanSA pocket-field models mirror key physical phenomena that are observed in protein-ligand interactions [15]: (1) choice of ligand poses is defined by the model; (2) non-additive (or even anti-additive) effects of substituent changes on a central scaffold can be modeled effectively; (3) changes in ligand structures induce changes in predicted ligand poses; and (4) the model of molecular activity is dependent on the detailed shape of ligands. Nearly all QSAR and deep-learning methods ignore some or all of these aspects of protein-ligand interactions. Additional discussion of the theoretical contrasts between the QuanSA multiple-instance learning approach and other QSAR (3D and 2D) approaches can be found in the papers introducing the method [11, 12] along with the antecedent QMOD [16] and Compass [17–19] approaches, the latter of which introduced the multiple-instance machine-learning paradigm [20].

Figure 2 depicts the overall scheme of the study. Beginning with the earliest 100 molecules and activity data (MET pIC_50_), a QuanSA model was induced, guided by a hypothesis of how a small set of diverse active ligands were mutually aligned. A set of “future” molecules that had been made on the way to (and including) Mol-1109 (the binding metabolite of florylpicoxamid: FPX) were then scored using the model. The scoring procedure predicts activity and bound ligand pose along with estimates of the degree to which each molecule is well-covered by the model. The top 10 molecules with *highest predicted activity* among those well-covered were selected for “synthesis.” In addition, the top 10 molecules expected to be *most informative* were also selected. Those 20 molecules were then used, along with their experimental activity values, to refine the model, moving those 20 from the test set to the train set, and this process was repeated (see the blue arrows in Fig. 2 for the refinement loop). The choice of informative molecules combines two criteria for a given molecule: (1) it must have a highly active training molecule as its nearest-neighbor in its QuanSA-predicted pose; and (2) it must be predicted to have relatively low activity. Simply put, the informative molecules are surprising: they look a lot like highly active molecules but are predicted to have poor activity.

In what follows, we show that the process of iterative model refinement drastically reduces the number of analogs required compared with what happened during the actual project. Successive models became progressively broader in terms of structural coverage and more accurate in their predictions. Separate from the activity prediction problem is the question of how one can generate synthetic candidate ideas that lead in a desired direction. We show how highly relevant analog ideas can be automatically generated using only a small number of compounds and potential pendant groups. The computational strategy presented here should have broad applicability in the common case where scaffold replacement is required and structure-activity data are limited and expensive to augment.

Software, computational protocols, and a subset of structure-activity data discussed in this paper are available to other researchers (see Declarations section).

## Results and discussion

We report results for iterative model refinement leading from the natural product antifungal UK-2A to FPX, beginning with a systematic procedure for identifying an informative multiple-ligand alignment and then proceeding through multiple rounds of QuanSA model refinement using an active learning strategy. We also detail a method to generate non-macrocyclic candidate compounds using very sparse data by combining virtual-screening-based central scaffold replacement with a simple method to “staple” appropriate substituents onto the replacement scaffolds.

**Fig. 3:**
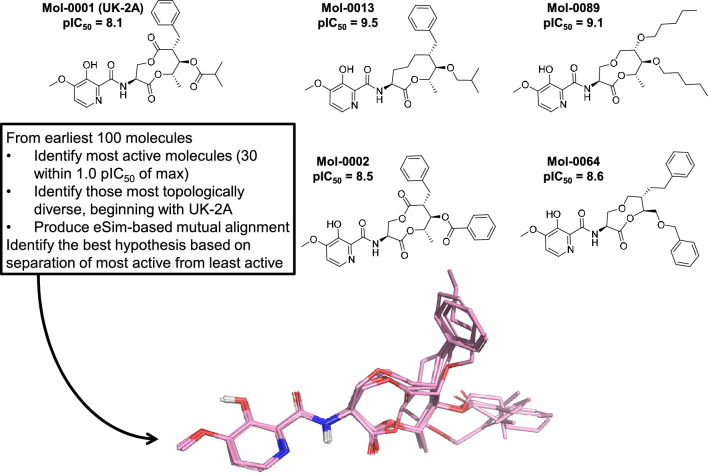
Procedure for identifying a high-quality ligand-based binding site hypothesis

### Initial multiple-ligand alignment

The QuanSA methodology derives a pocket-field beginning from an initial mutual alignment of a set of training ligands [11, 12], where each ligand has multiple possible initial poses. When protein structure information is available, it is possible to make use of the experimentally determined relative poses of prior known bound ligands in order to guide the construction of the initial set of training poses. Here, no such suitable protein co-crystal structure existed. Rather than using crystallographic data, it is also possible to make use of a carefully constructed multiple ligand alignment to guide model-building. In cases where scaffold diversity exists among highly active molecules, such alignments can provide significant constraints on the overall ligand alignment problem.

Here, the initial set of active project compounds contained significant diversity, both within the central macrocycle as well as in the pendant functionality. Figure 3 shows the procedure used to identify a high-quality ligand-based binding site hypothesis using only the data from the earliest set of synthesized molecules. There are two key ideas: (1) to identify structurally diverse active ligands from which to produce multiple ligands alignments; and (2) to select which of the alternative hypotheses of relative bound poses is quantitatively the best. The 30 molecules from within the top 1.0 log unit of experimentally determined activity among the training molecules were used as input to identify the four most 2D structurally diverse compounds (molecules 13, 89, 2, and 64 in Fig. 3). They were selected automatically based on 2D dissimilarity (see the “Methods and data” section for details).

These molecules (to the right of UK-2A in Fig. 3) differed in terms of size and flexibility within the central macrocycle as well as the composition of the right-hand substituents. They were used, with the addition of UK-2A, as input to the the multiple-ligand alignment functionality of the eSim method [21], which resulted in several alternative mutual superimpositions. In order to assess which mutual alignment was most likely to reflect the true relative poses of the molecules, the alternative alignments were ranked based on their ability to separate highly active molecules from relatively inactive ones within the initial 100-molecule training set. The chosen hypothesis shown in Fig. 3 was able to distinguish highly active (pIC$_{50} \ge 8.5$) from less active (pIC$_{50} \le 7.5$) compounds with an ROC Area of 0.92. The 3D joint superimposition shows the tight alignment of the common left-hand moiety (the “warhead”) with the variation in the macrocycle and right-hand elements of the molecules.

**Fig. 4:**
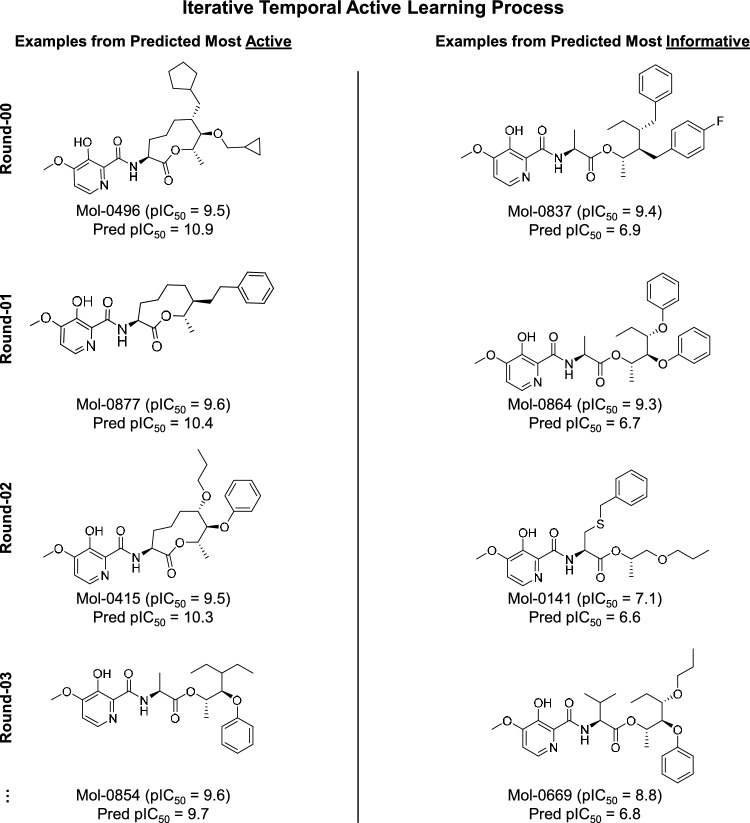
Example molecules chosen for model refinement in successive rounds of QuanSA testing and refinement

### Iterative model refinement

The chosen multiple-ligand alignment from Fig. 3 was used to guide construction of the initial QuanSA model pocket-field. The method allows for incremental iterative refinement based on the availability of new structure-activity data. Figure 4 shows examples of molecules automatically selected by QuanSA for model refinement based on expectations of high activity (left side) or based on expectations of being informative (right side) through multiple rounds of compound selection and model refinement. Intuitively, selection of candidate molecules based on predictions of high activity is an obvious strategy. In an active-learning paradigm, one also seeks to identify maximally *informative* molecules [22]. One representative example of each type of selection is shown for each of the first four rounds.

The process of scoring candidate molecules in a QuanSA pocket-field results in a prediction of activity and bound pose, along with a number of prediction quality metrics. The novelty metric characterizes the degree to which a candidate molecule is well-covered by the current set of training molecules. Candidate molecule predictions also indicate which training molecule was the nearest-neighbor (NN) in a 3D molecular similarity calculation based on the predicted bound pose.

Here, in each round, the 200 least novel (i.e. best covered) predicted candidate molecules were identified, and, of this subset, the top ten with highest predicted activity were selected for model refinement (see left-hand examples from Fig. 4). The maximally informative set of ten for each round captured a group of molecules that could be thought of as having unexpectedly low activity. Informative molecules were identified from the subset whose NN training molecule similarity was high (top 100 highest NN similarity *or* NN similarity $\ge$ 0.85) and where the NN training molecule’s activity was also high (pIC$_{50} \ge 8.5$). From that subset, the ten molecules with the lowest predicted activity were selected (see right-hand examples from Fig. 4).

**Fig. 5:**
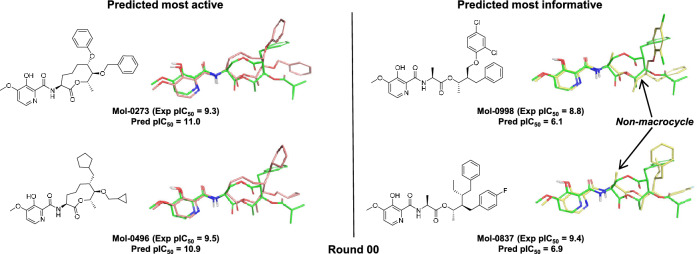
Selections of active (left, predicted poses in salmon carbons) and informative molecules (right, yellow carbons) for Round-00 shown against the predicted pose of UK-2A

In the early rounds, the compounds predicted to be highly active all had a central macrocyclic scaffold that was found among the most highly active training compounds, as would be expected given the starting point of lead optimization. However, after three rounds of model refinement (a cumulative addition of 60 molecules to the original model), a non-macrocycle was correctly identified and chosen as a highly active molecule (Fig. 4, lower left).

In contrast, the compounds predicted to be maximally informative included non-macrocycles even in the initial round of candidate selection. These compounds were deemed to be information rich: the predicted activities were low, yet these candidate molecules had very high 3D similarity to highly active train compounds. Model evolution through inclusion of these informative compounds broadened structural coverage sufficiently that a non-macrocycle was predicted to be highly active by Round-03 (bottom of Fig. 4).

#### Round-00: Initial model building and selection

QuanSA model building begins with an initialization step that produces training molecule alignments. Here, guided by the multiple-ligand alignment shown in Fig. 3, five alternative initial alignments were produced. Having been driven by the same mutual alignment hypothesis, these initial training molecule alignments differed only slightly, but each was used to build a separate QuanSA model. Selection from among alternative models can be done based on statistics derived from the alternative models. These include: (1) model parsimony, which is a quantitative measure of the extent to which molecules with similar activity values have similar predicted poses; (2) Kendall’s Tau for the full re-fitting of training molecules into a derived pocket-field; and (3) the mean unsigned error (MUE) of the re-fit molecules. The alternative quality values are transformed into probabilistic values, and their product reflects the combination of the different metrics. Here, the selected model exhibited a parsimony of 0.63, Kendall’s Tau of 0.87 (CI 0.82–0.91; p $< 10^{-4}$) and MUE of 0.30 (CI 0.25–0.35).

Figure 5 shows two representative examples from Round-00 for each selection type of candidate molecule. At left (salmon) are the predicted poses for two molecules among the ten predicted most active. As might have been expected, these test molecules have a macrocyclic scaffold in common with the most active training ligand. Also, the right-hand substituents largely occupy the same space as those of UK-2A. Although the activity predictions for compounds Mol-0273 and Mol-0496 were high, these molecules fell within the top 13% and 3%, respectively, of experimental activity within the full future set of 1009.

**Fig. 6:**
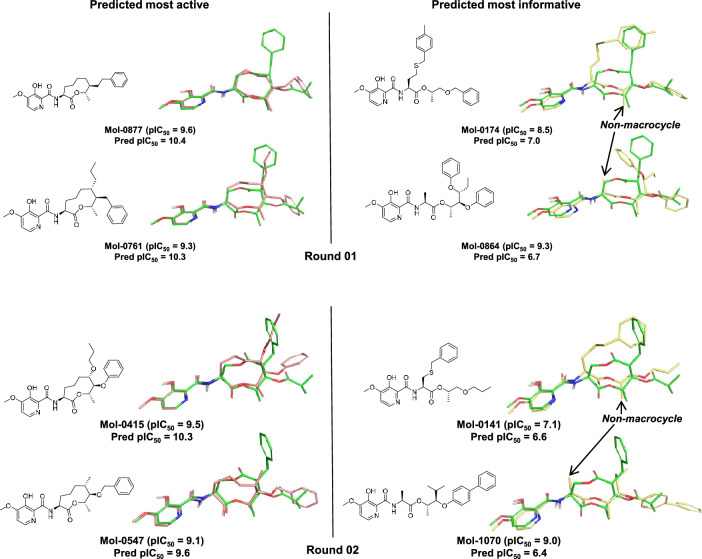
Selections of active and informative molecules for Round-01 and Round-02

At right (yellow) are the predicted poses of two molecules predicted to be among the ten most informative candidates. The poses of the test molecules are shown relative to the pose of training molecule UK-2A (green). These four examples are among the twenty molecules selected to refine the current training model. In contrast to the molecules chosen based on high predicted activity, the molecules chosen to be most informative in Round-00 included four non-macrocycles out of the ten chosen (two examples are shown in Fig. 5 at right). Importantly, the predicted 3D alignments compared with that of UK-2A (green) show the new scaffolds in tight congruence to the lower half of the UK-2A macrocycle. Also, the right-hand moieties of the informative molecules had significant surface overlap with those of UK-2A.

Overall, for the 10 predicted to be most active in Round-00, the MUE was quite high (1.7 pIC_50_ units), but, interestingly, these were all overpredictions. The predicted activity values exceeded even the maximal experimental activity of the most potent training molecule. This characteristic is not typically seen with traditional machine-learning approaches. With most statistical machine-learning methods and deep-learning methods, implicit or explicit modeling of the prior probability of observing a particular prediction value makes out-of-range predictions rare. This is a strength of moving toward a more causal type of predictive model where, for example, the combination of different aspects of multiple active molecules into a new candidate might lead to an out-of-range prediction. Particularly early-on in lead optimization, synthesis of candidate molecules that push the potency envelope is desirable.

**Fig. 7:**
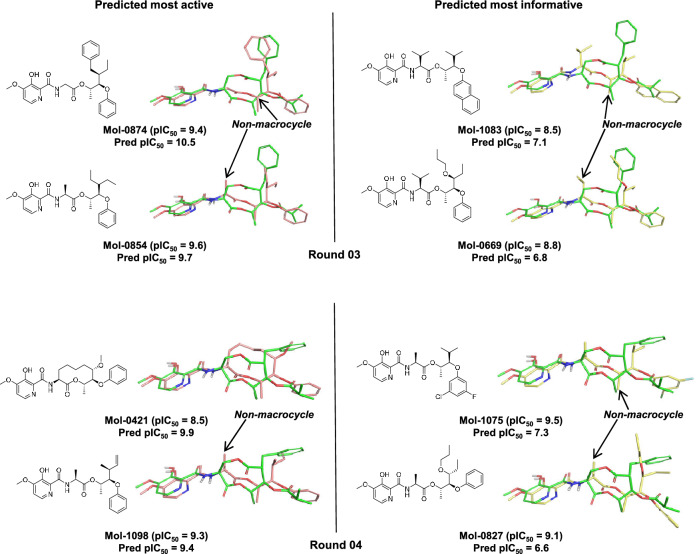
Selections of active and informative molecules for Round-03 and Round-04

#### Rounds 01-04: Refinement with active learning

Figure 6 shows examples of selected molecules for Round-01 and Round-02. Those compounds predicted to be most active retained macrocyclic scaffolds in both rounds, but they showed show some additional diversity in the right-hand hydrophobic groups, with alkyl chains aligning to the benzene moiety of UK-2A (see molecules Mol-0761 and Mol-0415). Also, the the nominal actives were more accurately predicted than for Round-00. For Round-01, the MUE was 1.4 pIC_50_ units. For Round-02, it was 0.9 pIC_50_ units, nearly 50% lower than for Round-00, indicating significant refinement in the detailed modeling of a subset of highly active ligands.

**Fig. 8:**
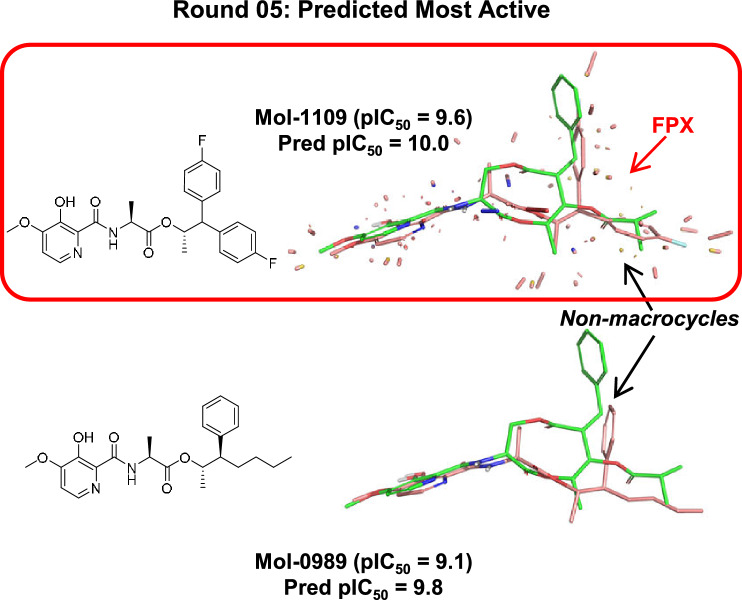
Selections of predicted active candidates for Round-05

Those candidates predicted to be most informative for rounds 01 and 02 contained a higher proportion of non-macrocycles that before. Round-01 had 7/10 non-macrocyclic candidate scaffolds, and Round-02 had 9/10 non-macrocyclic scaffolds (see examples Fig. 6, right, yellow). Alternative branching topologies were seen among the informative candidates as well as novel pendant groups. The flexible thio-ether linkages in compounds Mol-0174 and Mol-0141 still allowed the terminal aromatic rings to overlay well with the corresponding functionality of UK-2A.

One of the challenges in providing computational guidance for synthetic candidate prioritization is having a meaningful explanatory basis for predictions. As shown in Fig. 6, the optimal poses that come out of the fitting process into the quantitative pocket-fields offer convincing correspondence between predictions and known SAR. This is preferable to black-box predictions or those that may yield some explanatory information but do not provide a physically meaningful interpretation.

Figure 7 shows examples of the selected molecules for rounds 03 and 04. By Round-03, when only 60 previous future molecules had been used to refine the original 100-compound model, three non-macrocyclic scaffolds were among the ten predicted to be most active (two examples are shown: molecules Mol-0874 and Mol-0854). A non-macrocycle was also chosen in Round-04 among those predicted most active (Mol-1098, bottom left of Fig. 7). The trend of improvement in accuracy for the predicted most active candidates continued, with an MUE of 0.9 and 0.8 pIC_50_ units, respectively, for these two refinement rounds.

Those predicted most informative for Round-03 and Round-04 included 3/10 and 5/10 non-macrocycles. The decrease in the number of non-macrocycles in the informative set compared to Round-01 and Round-02 suggests that model refinement improved the predictions on non-macrocycles and thus these molecules would be less represented among those molecules that were being incorrectly predicted as having low activity.

Another aspect of quantitative activity prediction for this series was the clear importance of detailed hydrophobic shape on experimental activity. The ability of a compound to fill the presumed hydrophobic pockets of the non-warhead (right-hand) side of the binding site was a clear activity requirement. Accurately modeling such phenomena depends not only on a molecular representation that captures ligand shape, but also requires that predictions of molecular pose be respectful of the internal conformational energetics of candidate molecules.

#### Round-05: The goal compound

As shown in Fig. 8, FPX was chosen by the model that was trained on the initial 100 molecules and subsequently refined with 100 chosen based on the active learning strategy during the ensuing rounds of refinement. FPX was among the 10 predicted to be most active and the activity was accurately predicted with pIC$_{50} = 10.0$ with a signed error of just + 0.4 pIC_50_ units. The MUE of the 10 predicted to be most active was 0.8 pIC_50_ units, and importantly, the set included 7/10 non-macrocycles, evidence that the model had effectively learned the non-macrocyclic scaffold.

**Table 1:** Summary of rounds of model building and testing

Round	n Train	Train Tau	Train MUE	n Future	Future Tau	Future MUE	FPX Pred	FPX rank %
00	100	0.87 (0.82–0.91)	0.30 (0.25–0.35)	1009	0.35 (0.31–0.39)	1.24 (1.18–1.30)	7.3	61
01	120	0.84 (0.79–0.89)	0.34 (0.30–0.39)	989	0.35 (0.31–0.39)	0.95 (0.90–1.00)	7.6	62
02	140	0.85 (0.79–0.90)	0.32 (0.28–0.37)	969	0.41 (0.37–0.45)	0.85 (0.81–0.90)	8.5	36
03	160	0.82 (0.77–0.86)	0.36 (0.32–0.40)	949	0.40 (0.36–0.44)	0.76 (0.73–0.80)	8.5	44
04	180	0.82 (0.78–0.86)	0.35 (0.31–0.39)	929	0.38 (0.34–0.43)	0.78 (0.74–0.82)	8.8	26
05	200	0.82 (0.78–0.86)	0.34 (0.30–0.39)	909	0.46 (0.42–0.50)	0.70 (0.66–0.74)	10.0	1

Kendall’s Tau values are unitless and all had p $< 10^{-4}$. Mean unsigned error (MUE) and FPX predicted activity are in units of pIC_50_. Numbers in parentheses are 95% confidence intervals calculated by resampling with replacement

For FPX, in addition to the predicted pose, a depiction of the quantitative interactions with the pocket-field is shown in Fig. 8. The large majority of interactions were of a purely hydrophobic type, represented by salmon-colored sticks whose length is proportional to the interaction magnitude. Notably, the two fluoro-phenyl groups of FPX, which overlay corresponding hydrophobic functionality of UK-2A, are responsible for significant interactions. In addition, the two chiral methyl groups, especially the one at the lower right, were also important. Because of the angle needed to adequately display the key hydrophobic interactions, the specific polar interactions made by the warhead and the amide linker are somewhat difficult to discern, but all of the specific polar moieties were responsible for key interactions as well (blue and red sticks, for hydrogen-bond donors and acceptors, respectively). The other example highlights both the variability that can be tolerated in the pendant hydrophobic groups and the fact that the core scaffold shifts in accommodating different substituents.

In this *gedankenexperiment*, only 100 additional future molecules needed to be synthesized, tested, and added to the model in order to correctly choose FPX as an excellent candidate molecule. In completing Round-05, FPX and the other 19 chosen candidate molecules would be synthesized and tested. Overall, only 120/1009 future molecules needed to be “made” to both identify and confirm FPX as a highly active candidate with just two chiral centers, no macrocyclic component, and favorable synthetic characteristics.

### Temporal model evolution

Table 1 summarizes the statistics for the rounds of model building, refinement, and future predictions. The training re-fit Kendall’s Tau was consistently high (0.82–0.87) throughout the five rounds of refinement, indicating that model fidelity was maintained as new molecules were added. Likewise, the training re-fit MUE remained low (0.30–0.36 log units) throughout model refinement.

Here, the sets of future molecules were much larger than the training set, and they reflected substantial changes in the structural composition of molecules and the distribution of activity values compared with the training molecules. Later in the project, as expected, a larger proportion of synthesized molecules had very high activity. During successive rounds of scoring future molecules, Tau trended upward, increasing from 0.35 to 0.46. A large proportion of molecules had experimental activity values of 8.5–9.5. The small data range coupled with the presence of assay noise limits the upper bound on rank-based statistics.

More striking was the decrease in MUE for predictions on future molecules from 1.24 to 0.70. The model became significantly more accurate during refinement. As the future MUE decreased, the FPX predicted activity improved from pIC_50_ = 7.3 (signed error −2.2) in Round-00 to pIC_50_ = 10.0 (signed error +0.4) in Round-05. Model improvement was further reflected in the predicted rank of FPX activity which rose from the top 61% to the top 1%.

**Fig. 9:**
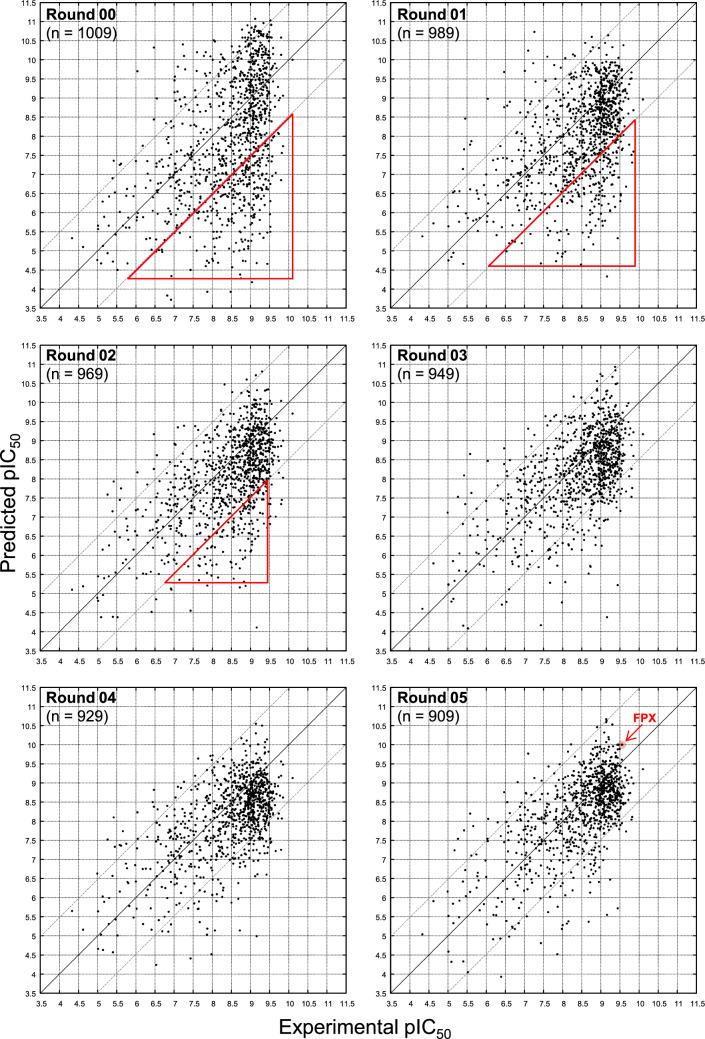
Experimental versus predicted activities for the set of all future molecules for each round of testing

**Table 2:** Summary of the distribution of predictions on future molecules

Round	n Train	n Future	% Pred w/in 1 kcal/mol	% Pred w/in 2 kcal/mol	% Underpredictions	% Overpredictions
00	100	1009	36	66	27	7
01	120	989	50	80	17	3
02	140	969	53	83	14	3
03	160	949	55	89	8	3
04	180	929	55	88	9	3
05	200	909	61	91	6	3

**Fig. 10:**
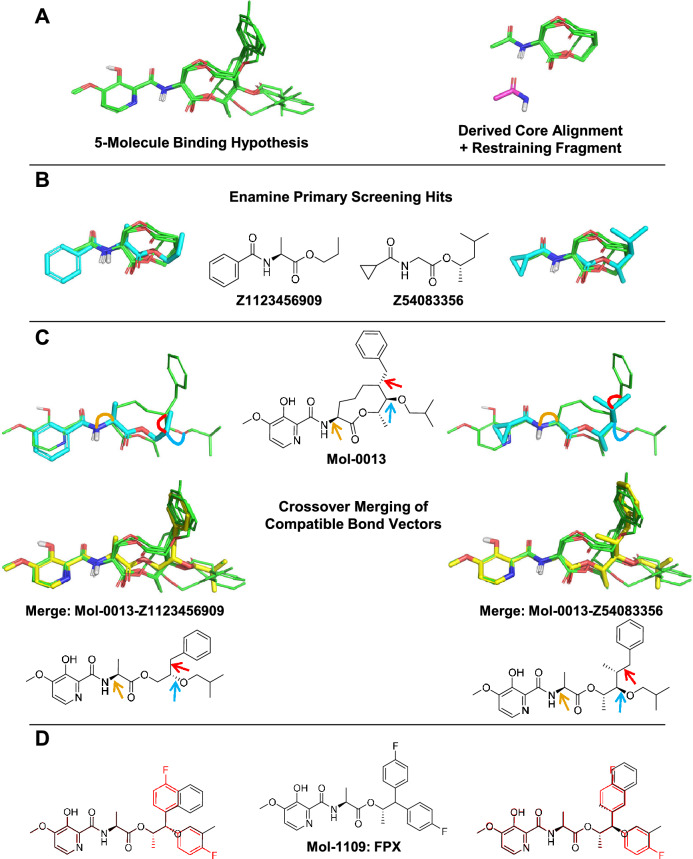
Scheme for generating synthetic ideas using a combination of eSim screening and automatic addition of desirable pendant groups

Figure 9 shows the plots of experimental versus predicted activities for the set of all future molecules for each round of testing. The identity line indicates perfect prediction, and the lighter lines represent $\pm 1.5$ units of pIC_50_ (corresponding to $\pm 2$ kcal/mol). The initial Round-00 model exhibited a strong lower-right triangular bias, with a significantly larger fraction of underpredictions than overpredictions. This aspect of the model’s predictive behavior shifted rapidly with the ensuing two rounds of active learning. By Round-03, relatively little skew was apparent. The distribution of underpredictions (< −2 kcal/mol, Fig. 9, red triangles) decreased 10 percentage points from Round-00 to Round-01 and became nearly as few as the overpredictions in Round-03 to Round-05. The Round-05 FPX prediction (Exp pIC_50_ = 9.5, Pred pIC_50_ = 10.0) is highlighted in red.

Table 2 shows a summary of the distribution of predictions on future molecules depicted in the plots of Fig. 9. Throughout model refinement and predictions on future molecules, large overpredictions were few and relatively constant (7% in Round-00 to 3% in Round-05). The predictions within 2 kcal/mol increased from 66% in Round-00 to 91% in Round-05, and those within 1 kcal/mol from 36% in Round-00 to 61% in Round-05. The dramatic decrease in underpredictions occurred in two steps, from Round-00 (27%) to Round-01 (17%) and from Round-02 (14%) to Round-03 (8%). By Round-05, the fractions of large over- and under-predictions were essentially the same.

The distribution of experimental activity values for the future set of molecules changed relatively little over time, perhaps as expected given that only roughly 10% of the molecules were selected over the rounds of iterative refinement. The minimum and maximum pIC_50_ values were 4.3 and 10.1, respectively, throughout. The mean and standard deviation began with $8.5\pm 1.0$ and ended with $8.4\pm 1.0$. However, for the *training set*, the distribution shifted. The initial minimum and maximum pIC_50_ training values were 4.3 and 9.5, respectively, shifting to 4.3 and 9.9 at the end of refinement. The mean and standard deviation began with $7.6\pm 1.3$ and ended with $8.1\pm 1.2$. The distributional shifts in the training data during refinement reflected the successful selection of numerous potent candidate molecules.

### Idea generation

One difficulty in interpreting the results shown in the foregoing is that the set of molecules from which we selected molecules had been made and tested as part of an active design process, where decisions on what to make next were undertaken by experts based on their knowledge of prior data as well as their expertise in the field. So, while the active learning approach was able to efficiently select from *that* set of molecules, it is not clear that such a path could be followed in a situation where the future space of molecules was open to determination.

Generative approaches for producing ideas for new compounds that employ deep learning have gained some prominence recently [9, 10]. We have taken a different approach, instead using molecular similarity to identify possible bioisosteric core scaffold replacements, including their suitability to display the require pendant functionality for good activity. Figure 10 illustrates how a combination of similarity-based screening and combination with desirable pendant groups can rapidly generate ideas. Our approach is similar in spirit to work by Awale, Hert et al. [23], in which the authors describe a 2D matched-pair approach to identifying sensible candidate molecules based on an analysis of large structure-activity databases.

Beginning with the original five-molecule multiple-ligand alignment used to guide QuanSA model-building, the pendant groups were removed to produce a core-scaffold overlay, and the amide linking subfragment was extracted (Fig. 10A at right). The roughly 3,000,000 compound Enamine Stock Screening collection was screened against the multiple-ligand core using the amide fragment as a required positional restraint to ensure that all hits returned would have appropriate chemistry for linkage to the common warhead. Two examples of high-scoring hits from the screen are shown in Fig. 10B (cyan carbons) in their optimal poses relative to the screening target (green carbons).

For each returned pose of each nominal screening hit, a geometric matching procedure was employed to identify crossover points between the screening hit and each of the full parent molecules from the original multiple-ligand alignment. Figure 10C shows the process using the pose of compound Mol-0013 as the crossover target. When a compatible set of distances and bond vectors existed, the original substituents of the screening hit were replaced with the substituents of the parent compound. Figure 10C (bottom) shows the two resulting merged molecules with novel structures. The arrows and corresponding thick lines show the specific substituent movements that were made. The initial crossover results in high local strain for the new bonds, and the final ligand pose is relaxed using positionally-restrained energy minimization.

Figure 10D shows the relationship of the two resulting generated candidate molecules. Each contains a large fraction of the exact substructure (including chirality) of the final FPX compound, with relatively minor variations in the precise hydrophobic substituents at right. With a slight generalization of the procedure to include additional substituent variations (e.g. p-fluoro-phenyl at both positions), the exact structure of FPX would have been generated. The data required to identify the five-molecule multiple-ligand alignment was just the first 100 compounds from the full structure-activity set. The computational procedure for identifying core-scaffold hits and producing merged candidate molecules required less than an hour and *no additional data*.

The procedure just described is not intended to fully automate candidate compound generation. Rather, it is meant to be a source of ideas that are easy to scan rapidly. Of course, it is also possible to make use of the predictive QuanSA models to identify candidates that are quantitatively predicted to have high potency or have high information value.

## Conclusions

Overall, beginning with the earliest 100 picolinamide antifungal project compounds, an active-learning approach efficiently guided candidate selection to the desired end product FPX after model refinement using just 100 synthetic analogs. This project began with a relatively potent lead compound in UK-2A, with design goals including a reduction in molecular complexity that required replacement of the central macrocyclic scaffold. This presents a challenge for predictive modeling because the molecules to be designed *must* deviate quite significantly from known chemical matter. Through the use of active learning, rapid introduction of novel structural features was possible. The process was guided by a well-defined notion of what makes a highly informative molecule—one that exhibits high similarity to a known active in their respective optimal predicted poses but which is (possibly anomalously) predicted to have low activity.

The practical significance of the restrospective analysis presented here is in the breadth of applicability for scaffold replacement and lead optimization more broadly. The QuanSA method does not require a protein structure to make accurate predictions that are physically explainable. While it can make use of information from experimental determination of bound ligand structures, it can operate in a purely ligand-based manner where the only available data are compound structures and activities. Model building can proceed from very limited project data, beginning with just dozens of molecules, not the thousands required for so-called deep-learning methods [9, 10].

Further, model-building is not terribly computationally intensive. On modest workstation hardware, candidate molecules can be scored in seconds for “normal” small molecules. Small macrocycles such as those seen here required tens of seconds per molecule, with a majority of the time going to conformational search. For the work reported here, the fully-automated procedure took approximately two days on an 18-core workstation. This encompassed the entire process beginning with 2D structures for all 1109 molecules, through model-building, scoring/selection/refinement, and the final pass from which FPX was chosen.

The lead optimization project that resulted in FPX required the synthesis of many hundreds of analogs in order to re-engineer the starting macrocyclic natural product. We believe that effective use of active learning and semi-automatic candidate generation can drastically shorten the design path from initial lead compound to final product. The central requirement for the computational methodology is that it is capable of extrapolating from small quantities of structure-activity data. Modeling approaches that move toward constructing causal models for activity prediction have clear advantages over approaches that ignore the physical underpinnings of how ligands bind to and modulate the activity of biological targets.

## Methods and data

### Molecular data set

A total of 1109 compounds from a lead optimization project formed the data set. Molecules were provided as 2D SDF structures with associated activities and consecutive compound IDs serving as relative synthesis dates for temporal sorting. The project dataset contained pIC_50_ activity values and registration dates, beginning with UK-2A as compound 1 and the resultant commercial product FPX as compound 1109. The activity values were determined in an in vitro assay for the inhibition of fungal mitochondrial electron transport. The first 100 molecules synthesized were used as the initial training set for QuanSA, with the remaining 1009 molecules used as future “synthesizable” molecules.

### Computational procedures

For all procedures, we employed version 5.1 of the Surflex Platform (BioPharmics Division, Optibrium Limited, Cambridge, CB25 9GL, UK). Additional details can be found in the data archive associated with this paper (see the Declarations section).

#### Ligand preparation

Standard procedures were used to protonate the molecules as expected at physiological pH, generate 3D structures, and perform conformational search, as follows:
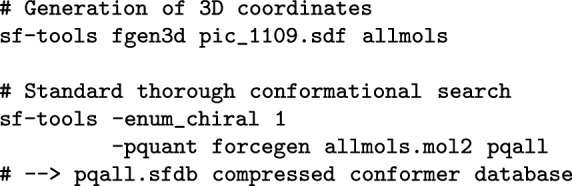


#### Multiple ligand alignment

Using Surflex eSim for generating multiple ligand alignments [21], and specifically for the purpose of seeding initial QuanSA alignments was outlined earlier and has been reported previously [11], with the specific procedure used in this work being as follows:
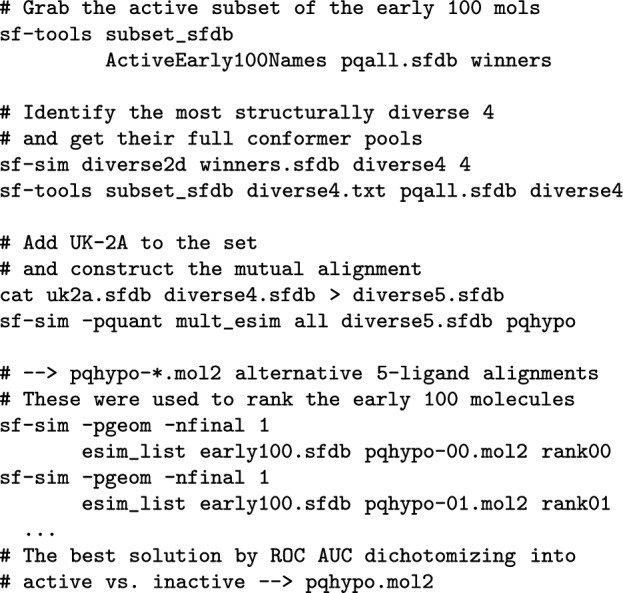


#### QuanSA model induction, prediction, and refinement

Previous QuanSA method papers are comprehensive, and contain a detailed algorithmic description [11, 12]. Here, standard procedures were used, as follows:
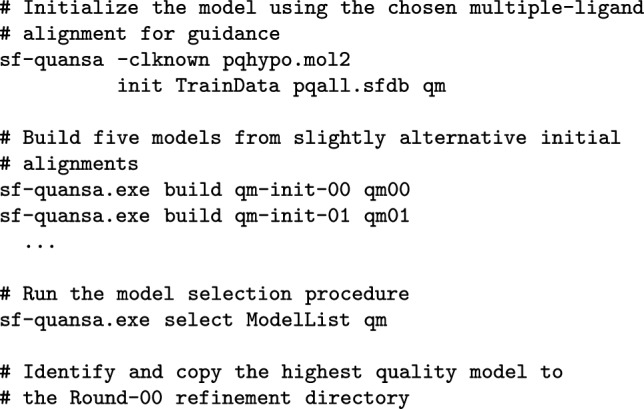


The model selection procedure select command generates a model quality score that combines the following: (1) model parsimony (P), which is a quantitative measure of the extent to which molecules with similar activity values have similar predicted poses; (2) Kendall’s Tau (T) for the full re-fitting of training molecules into a derived pocket-field; and (3) the mean unsigned error (E) of the re-fit molecules.

Given N alternative models, each of $P_{1...N}$, $T_{1...N}$, and $E_{1...N}$ are transformed into corresponding probability scores. This is done by fitting a normal distribution to each of $P_{1...N}$, $T_{1...N}$, and $E_{1...N}$ which then allows calculation of the cumulative distribution function $\Phi$ for each of *P*, *T*, and *E*. So, raw values for the metric across the N alternative models are converted to probabilities reflecting their likelihood of being non-random: $P_{1...N}^p$, $T_{1...N}^p$, and $E_{1...N}^p$. The probability score for model *i* is simply the product: $(P_i^p)(T_i^p)(E_i^p)$. The highest scoring of the alternative models using the combined probabilistic score is selected.

New molecule scoring, selection, and model refinement followed these general procedures:
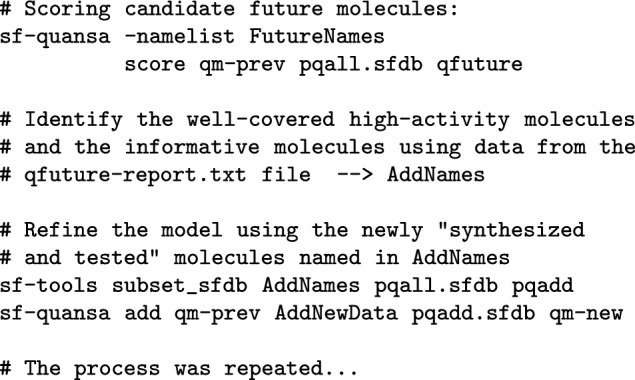


#### Computational procedures for idea generation

The pendant groups were trimmed from the 5-molecule multiple ligand alignment described above, leaving only the aligned central scaffolds. The aligned core scaffolds were used as a multi-ligand target in a virtual screen of the Enamine database. The resulting hits were processed using a new procedure to automatically attach pendant groups from the original full ligands of the multiple-ligand alignment, as follows:
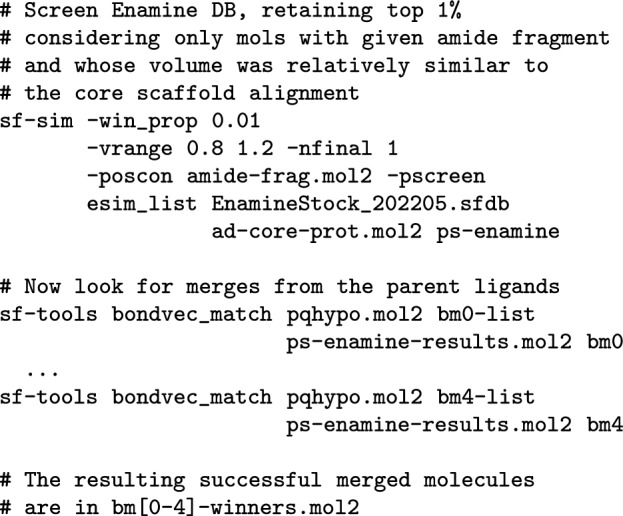


Note that the resulting merged molecules can be reviewed directly or can be subjected to conformational search and screened using either pure ligand similarity, QuanSA model scoring, or a combination of both approaches.

## Data Availability

A freely downloadable data archive with additional details is available at www.jainlab.org/downloads. The archive contains scripts to reproduce the protocols described here beginning from 2D input structures. Note that the compound structures in the archive are limited to those depicted here and do not include the full set of 1109 ligands described, whose structures and activity values cannot be disclosed.

## References

[1] Meyer KG, Bravo-Altamirano K, Herrick J, Loy BA, Yao C, Nugent B, Buchan Z, Daeuble JF, Heemstra R, Jones DM, Wilmot J, Lu Y, DeKorver K, DeLorbe J, Rigoli J (2021) Discovery of florylpicoxamid, a mimic of a macrocyclic natural product. Bioorg Med Chem 50(116):455 10.1016/j.bmc.2021.116455 34757295

[2] Labute P (2010) LowModeMD: implicit low-mode velocity filtering applied to conformational search of macrocycles and protein loops. J Chem Info Model 50(5):792–800 10.1021/ci900508k 20429574

[3] Chen IJ, Foloppe N (2013) Tackling the conformational sampling of larger flexible compounds and macrocycles in pharmacology and drug discovery. Bioorg Med Chem 21(24):7898–7920 24184215 10.1016/j.bmc.2013.10.003

[4] Watts KS, Dalal P, Tebben AJ, Cheney DL, Shelley JC (2014) Macrocycle conformational sampling with MacroModel. J Chem Inf Model 54(10):2680–2696 25233464 10.1021/ci5001696

[5] Sindhikara D, Spronk SA, Day T, Borrelli K, Cheney DL, Posy SL (2017) Improving accuracy, diversity, and speed with prime macrocycle conformational sampling. J Chem Info Model 57(8):1881–1894 10.1021/acs.jcim.7b00052 28727915

[6] Cleves AE, Jain AN (2017) ForceGen 3D structure and conformer generation: From small lead-like molecules to macrocyclic drugs. J Comput Aided Mol Des 31(5):419–439 28289981 10.1007/s10822-017-0015-8 PMC5429375

[7] Jain AN, Cleves AE, Gao Q, Wang X, Liu Y, Sherer EC, Reibarkh MY (2019) Complex macrocycle exploration: parallel, heuristic, and constraint-based conformer generation using ForceGen. J Comput Aided Mol Des 33(6):531–558 31054028 10.1007/s10822-019-00203-1 PMC6554267

[8] Jain AN, Brueckner AC, Jorge C, Cleves AE, Khandelwal P, Cortes JC, Mueller L (2023) Complex peptide macrocycle optimization: combining NMR restraints with conformational analysis to guide structure-based and ligand-based design. J Comput Aided Mol Des 37:519–535 37535171 10.1007/s10822-023-00524-2 PMC10505130

[9] Walters WP, Barzilay R (2020) Applications of deep learning in molecule generation and molecular property prediction. Acc Chem Res 54(2):263–270 33370107 10.1021/acs.accounts.0c00699

[10] Ramsundar B, Eastman P, Walters P, Pande V (2019) Deep learning for the life sciences: applying deep learning to genomics, microscopy, drug discovery, and more. O’Reilly Media Inc, Newton

[11] Cleves AE, Jain AN (2018) Quantitative surface field analysis: learning causal models to predict ligand binding affinity and pose. J Comput Aided Mol Des 32(7):731–757 29934750 10.1007/s10822-018-0126-x PMC6096883

[12] Cleves AE, Johnson SR, Jain AN (2021) Synergy and complementarity between focused machine learning and physics-based simulation in affinity prediction. J Chem Inf Model 61(12):5948–5966 34890185 10.1021/acs.jcim.1c01382

[13] Jain AN (1996) Scoring noncovalent protein-ligand interactions: a continuous differentiable function tuned to compute binding affinities. J Comput Aided Mol Des 10(5):427–440 8951652 10.1007/BF00124474

[14] Pham T, Jain AN (2006) Parameter estimation for scoring protein-ligand interactions using negative training data. J Med Chem 49(20):5856–5868 17004701 10.1021/jm050040j

[15] Jain AN, Cleves AE (2012) Does your model weigh the same as a Duck? J Comput Aided Mol Des 26:57–67 22187141 10.1007/s10822-011-9530-1 PMC3276372

[16] Cleves AE, Jain AN (2016) Extrapolative prediction using physically-based QSAR. J Comput Aided Mol Des 30(2):127–152 26860112 10.1007/s10822-016-9896-1 PMC4796382

[17] Jain AN, Dietterich TG, Lathrop RH, Chapman D, Critchlow REJ, Bauer BE, Webster TA, Lozano-Perez T (1994) A shape-based machine learning tool for drug design. J Comput Aided Mol Des 8(6):635–52 7738601 10.1007/BF00124012

[18] Jain AN, Koile K, Chapman D (1994) Compass: predicting biological activities from molecular surface properties. Performance comparisons on a steroid benchmark. J Med Chem 37(15):2315–27 8057280 10.1021/jm00041a010

[19] Jain AN, Harris N, Park J (1995) Quantitative binding site model generation: compass applied to multiple chemotypes targeting the 5-HT1a receptor. J Med Chem 38(8):1295–1308 7731016 10.1021/jm00008a008

[20] Dietterich TG, Lathrop RH, Lozano-Pérez T (1997) Solving the multiple instance problem with axis-parallel rectangles. Artif Intell 89(1–2):31–71

[21] Cleves AE, Jain AN (2020) Structure-and ligand-based virtual screening on DUD-E^+^: performance dependence on approximations to the binding pocket. J Chem Inf Model 60(9):4296–4310 32271577 10.1021/acs.jcim.0c00115

[22] Varela R, Walters W, Goldman B, Jain AN (2012) Iterative refinement of a binding pocket model: active computational steering of lead optimization. J Med Chem 55(20):8926–8942 23046104 10.1021/jm301210j PMC3640415

[23] Awale M, Hert J, Guasch L, Riniker S, Kramer C (2021) The playbooks of medicinal chemistry design moves. J Chem Inf Model 61(2):729–742 33522806 10.1021/acs.jcim.0c01143

